# Prospective surveillance of colonization and disease by methicillin-resistant *Staphylococcus aureus* (MRSA) at a European pediatric cancer center

**DOI:** 10.1007/s00520-022-07140-0

**Published:** 2022-05-19

**Authors:** Miriam A. Füller, Stefanie Kampmeier, Anna M. Wübbolding, Judith Grönefeld, Almut Kremer, Andreas H. Groll

**Affiliations:** 1 Infectious Disease Research Program, Center for Bone Marrow Transplantation and Department of Pediatric Hematology and Oncology, University Children’s Hospital Münster, Münster, Germany; 2 Institute of Hygiene, University Hospital Münster, Münster, Germany; 3 Medical Controlling, University Hospital Münster, Münster, Germany

**Keywords:** MRSA, Colonization, Infection, Children, Cancer, Transplantation

## Abstract

**Purpose:**

Children and adolescents undergoing treatment for cancer or allogeneic hematopoietic cell transplantation are at increased risk for methicillin-resistant *Staphylococcus aureus* (MRSA). We therefore examined the occurrence and outcome of MRSA colonization and infection in patients of a large European pediatric cancer center.

**Methods:**

In a prospective observational cohort study conducted between 2007 and 2018, nasopharyngeal swabs for culture of MRSA were obtained from all admitted patients. The primary endpoint of the study was the colonization rate over time. Secondary endpoints included genetic relatedness of isolates, time burden of isolation measures, and results of decolonization efforts.

**Results:**

During the study period, MRSA screening identified 34 colonized patients (median age: 10 years; range: 0–21) without trends over time. MRSA colonization was associated with the presence of classical risk factors. There was no molecular evidence of patient-to-patient transmission. A standard MRSA eradication regimen led to a lasting eradication of the organism in 26 of 34 patients. MRSA infection occurred in two patients with no associated fatalities.

**Conclusion:**

Prospective monitoring revealed low rates of MRSA colonization and infection at our center. These low rates and the absence of patient-to-patient transmission support the effectiveness of the management bundle of MRSA identification, isolation, and decolonization.

**Supplementary Information:**

The online version contains supplementary material available at 10.1007/s00520-022-07140-0.

## Introduction

Colonization and subsequent infection by methicillin-resistant *Staphylococcus aureus* (MRSA) is a major global health issue. While between 1 and 3% of all hospitalized patients in Germany are colonized by MRSA, individual European countries and the USA report higher prevalence rates [1–3]. Children and adolescents undergoing treatment for cancer and/or allogeneic hematopoietic cell transplantation (HCT) represent a particularly vulnerable patient population as they usually carry several established risk factors for MRSA colonization, including indwelling central venous catheters, frequent administration of broad-spectrum antibacterial agents, frequent hospitalization, and surgical interventions [4]. Additionally, MRSA is the only antibiotic-resistant pathogen in which previous colonization was significantly associated with a higher risk of invasive infections in pediatric patients receiving chemotherapy or HCT [5]. MRSA infections in critically ill and immunocompromised children and granulocytopenia and immunosuppressive treatment may lead to rapidly progressive and potentially fatal infections [6, 7]. Since acquisition of MRSA colonization during a hospital stay has been identified as the highest risk factor for subsequent infection, prevention of nosocomial MRSA transmission remains a cornerstone in the prevention of invasive disease [6]. However, in pediatric patients with cancer or undergoing allogeneic HCT, very limited data exist regarding the exact role and the impact of MRSA decolonization for prevention of MRSA infection, the effectiveness of patient isolation to avoid nosocomial transmission and of infection control bundles to achieve decolonization, and the question of whether MRSA colonization has to be considered for the choice of the initial empiric antibiotic regimens in case of fever during granulocytopenia.

In order to control MRSA transmission and emergence of MRSA infections among pediatric patients with cancer and allogeneic HCT in the hospital setting, we implemented mandatory screening, isolation, and decolonization in all inpatient admissions from 2007 onward. Here we report the results of an audit conducted between 2007 and 2018 to analyze the prevalence and outcome of MRSA colonization and infection in this vulnerable patient population with emphasis on the efficacy of MRSA decolonization and nosocomial transmission in immunocompromised patients.

## Patients and methods

### Study design overview

In a prospective, single center observational cohort study, MRSA colonization, nosocomial transmission, decolonization success, and occurrence of invasive disease were analyzed in all pediatric patients admitted to the pediatric hematology/oncology inpatient ward and the inpatient unit of the Center for Bone Marrow Transplantation between January 2007 and December 2018. Written informed consent for hemato-oncological and supportive care was obtained from participants if participants were 18 years or older and from participants’ parents or legal guardians if participants were younger than 18 years within the consent procedure for cancer treatment, HCT, and specialized medical care. Data collection was accomplished by a pseudonymized standardized case report form. The primary endpoint was the MRSA colonization rate over time as assessed by mandatory inpatient admission screening implemented in 2007. Secondary endpoints included resistance profiles, MRSA spa-types, and genetic distribution of colonizing MRSA isolates; assessment of clinical risk factors for MRSA colonization; implementation and effectiveness of MRSA decolonization; and total days spent on isolation as inpatients. Occurrence and outcome of invasive MRSA infections were also assessed. All clinical and microbiological data were tabulated and analyzed using descriptive statistics unless stated otherwise.

### Clinical setting

The Department of Pediatric Hematology and Oncology of the University Children’s Hospital of Münster serves a catchment area of five million people in the Northwest of Germany. Each year, 140–160 unselected patients with a new diagnosis of cancer and 20–40 patients with the diagnosis of recurrent cancer are admitted. The Department maintains a HCT program accredited by the Joint Accreditation Committee of the International Society for Cellular Therapies and the European Society for Blood and Marrow Transplantation (JACIE) with approximately 30 allogeneic and 10 autologous HSCT procedures performed per year [8]. During the study period, the number of inpatient admissions per year was between 1000 and 1188, accounting for between 5165 and 5973 inpatient days per year. These admissions also included a small (< 1%) percentage of non-transplanted patients with benign hematological disorders receiving inpatient supportive care. Except for patients undergoing allogeneic HCT (reverse isolation in HEPA-filtered single bed rooms) and those requiring contact precautions and isolation (regulated by a standardized manual maintained and updated by Infection Control), all patients were cared for in open, two- or four-bed patient rooms with 24-h presence of a caretaker. As standard of care, patients received non-absorbable polyenes for prevention of mucosal candidiasis and trimethoprim/sulfamethoxazole (post-engraftment in HCT recipients) twice weekly for prevention of *Pneumocystis jirovecii* pneumonia. Routine antibacterial prophylaxis was not administered apart from HCT patients who, until 2014, received penicillin, ciprofloxacin, and metronidazole from admission through occurrence of the first fever. Patients with leukemia or those undergoing allogeneic HCT received azole- or polyen-based antifungal prophylaxis [9, 10]. Ceftazidime plus gentamicin (until 2014) and piperacillin-tazobactam (thereafter; plus gentamicin only in allogeneic HCT recipients) were used as initial regimen for empiric antibacterial therapy, and meropenem plus glycopeptide in critically ill or unstable patients and those with persistent or recurrent fever for empirical escalation.

### Infection control measures

The facilities of the University Children’s Hospital are integrated into the patient care facilities of the University Hospital of Münster, a 1500-bed tertiary care center delivering inpatient care for approximately 61,000 separate inpatient admissions per year [11]. Since 2007, all inpatients are screened upon each in-hospital admission for MRSA according to an internal standard operation procedure and more recently published national guidelines [12] by a combined nasal and pharyngeal swab culture, and a swab culture from chronic wounds, if present. Caretakers of pediatric patients identified to be colonized and of those patients admitted to the inpatient unit of the center for bone marrow transplantation are also screened as precaution or to facilitate and synchronize decolonization, respectively. In case of MRSA detection, combined axillary and combined inguinal swabs are added, and extended hygiene measures are implemented per standard operating procedure. These include contact isolation in a separate room and strict separation of sanitary facilities. All visitors and staff members are instructed to wear personal protective equipment when entering a patient room, consisting of gloves, gowns, and surgical masks. MRSA screening for healthcare workers is not routinely performed. Surface cleaning disinfection is performed once a day. Patients are de-isolated, if MRSA-negative status is confirmed after decolonization. The decolonization protocol includes nasal ointment with mupirocin 2% three times daily, gargling with octenidin (Octenidol®; Schülke & Mayr, Norderstedt, Germany) three times daily and washing skin and hair with octenidin (Octenisan® Schülke & Mayr, Norderstedt, Germany) once per day. This treatment is performed for 5 days total. Three days after the last treatment, screening is performed daily for 3 days to test the success of the decolonization by combined swabs obtained each from the nasopharynx, the axillary, and the inguinal regions and infected wounds, if present. In accordance with national guidelines [12], long-term effectiveness of decolonization is assumed, if screening samples remain MRSA-negative during a 12-month period after first MRSA decolonization.

### MRSA sampling, culture techniques, and antibacterial resistance and PCR testing

MRSA screening by nasopharyngeal samples was performed using Polywipe™ (Medical Wire & Equipment, Wiltshire, United Kingdom) pre-moistened sponge swabs. Detection of MRSA was performed by using selective agar plates (chromID®, bioMérieux, Marcy l’Étoile, France) and incubation at 35 ± 1 °C for 24–48 h both with and without enrichment (Dextrose Bouillon) at 36 °C for 24 h. Suspicious colonies were confirmed via MALDI-TOF–MS (Bruker Corporation, Bremen, Germany). Susceptibility testing was performed and interpreted in accordance with the current European Committee on Antimicrobial Susceptibility Testing (EUCAST) standards for clinical breakpoints [13]. Methicillin resistance was confirmed by the PBP2a latex agglutination test (PBP2a SA Culture Colony Test®, Abbott, Illinois, US) and genotypically by detecting the *mecA*, *mecC resistance*, and the Panton-Valentine leukocidin (PVL) virulence genes (Geno-Type MRSA®, HAIN, Nehren, Germany) [14, 15].

### Whole genome sequence-based typing (WGS)

In order to detect potential nosocomial transmissions via genetic similarities of isolated strains, the protein A gene (spa) polymorphism in MRSA isolates were implemented into surveillance routine before 2013. Since 2013, whole genome sequencing approaches of all detected multi-resistant bacteria are established in order to determine the genetic relatedness of MRSA and providing a higher resolution than spa-typing [16]. MRSA isolates were compared genetically via WGS using the Illumina NextSeq® and MiSeq® platforms (Illumina Inc., San Diego, CA, USA). With the help of the SeqSphere + ® software (version 6.0.0), coding regions were compared in a gene-by-gene approach (core genome multilocus sequence typing, cgMLST) and clonal relationships were visualized via a minimum spanning tree. Genotypes that differed in ≤ 24 alleles were defined as being genetically related. For backwards compatibility with classical molecular typing, spa-types and for detection of PVL encoding genes *lukS* and *lukF* were extracted from the WGS data in silico.

## Results

### MRSA colonization rates and patient characteristics

**Fig. 1:**
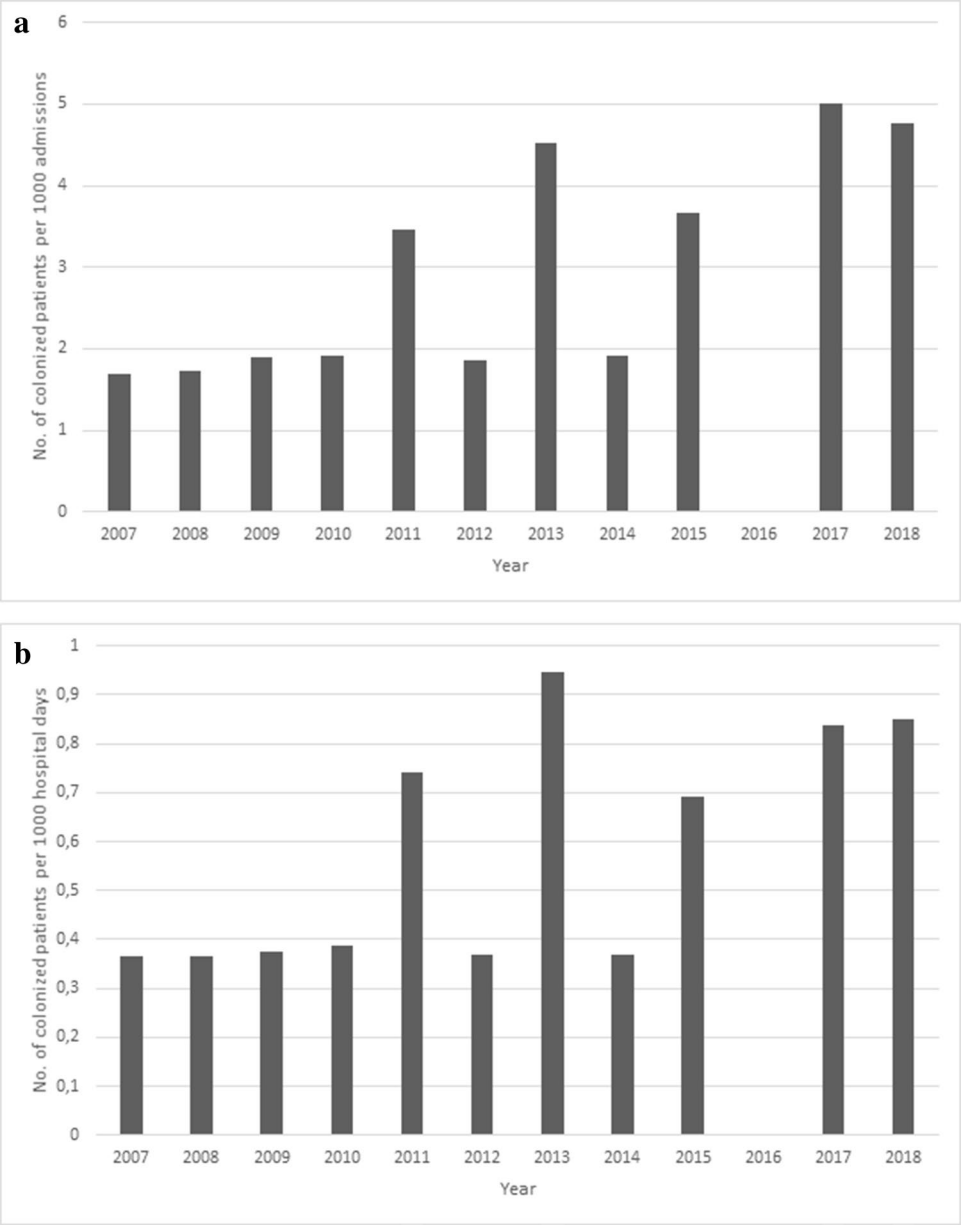
MRSA colonization rates in pediatric patients with cancer or allogeneic HCT over time. Depicted are the number of colonized patients per 1000 inpatient admissions per year (**a**) and the number of colonized patients per 1000 hospital days per year (**b**). Each colonized patient was counted as a case only once at the time of the initial presentation. Comparison of colonization rates during 2007–2012 and during 2013–2018 by contingency tables and two-tailed chi-square tests with Yate’s correction revealed no differences between the two time periods (*p* = 0.1770 and *p* = 0.2898, respectively). In 2016, no patient with MRSA colonization was admitted; only one parent (P30) was tested positive

From January 2007 until end of December 2018, of 1912 patients, 34 patients were identified with a new diagnosis of MRSA colonization by routine screening at the time of inpatient admission. Colonization rates over time during the 12 years of observation ranged from 0 to 5 per 1000 inpatient admissions per year and were between 0 and 0.94 per 1000 inpatient days per year. Comparison of colonization rates during 2007–2012 and during 2013–2018 revealed no differences between the two time periods (*p* = 0.1770 and *p* = 0.2898, respectively) (Fig. 1). In order to monitor adherence to routine inpatient admission screening, screening rates were randomly assessed during two different 3 months time periods (November 2012–January 2013 and October 2018–December 2018). Adherence rates during the selected periods were 94% (211/235 admissions) and 73% (194/267admissions), respectively, suggesting acceptable compliance but also the need of regular monitoring.

The median age of the 34 patients was 10.3 years (range: 0–21) and most were of male gender (24; 70.6%) (Table S1). Underlying conditions were acute leukemia (12) or Hodgkin’s lymphoma (1), solid tumors (10), brain tumors (4), non-malignant hematological disorders (6), and cerebral vasculitis (1). Established risk factors for MRSA colonization included the presence of a central venous catheter (26; 76%), antibiotic treatment in the previous four weeks (18; 53%), status post recent surgery (14; 41%), migration background (11; 32%), and previous allogeneic hematopoietic cell transplantation (11; 32%). While all patients had positive swabs from nose and throat, swabs from the axilla and/or groins were positive for MRSA in nine of 27 patients tested (33%). In four of the affected patients, at least one parent (five in total) was found to be colonized by MRSA, and three more parents were tested positive for MRSA prior to admission of their children to the transplant unit. However, not all parents of colonized patients have been examined (Table S1).

### Resistance profiles, MRSA spa-types, and genetic distribution of strains

**Fig. 2:**
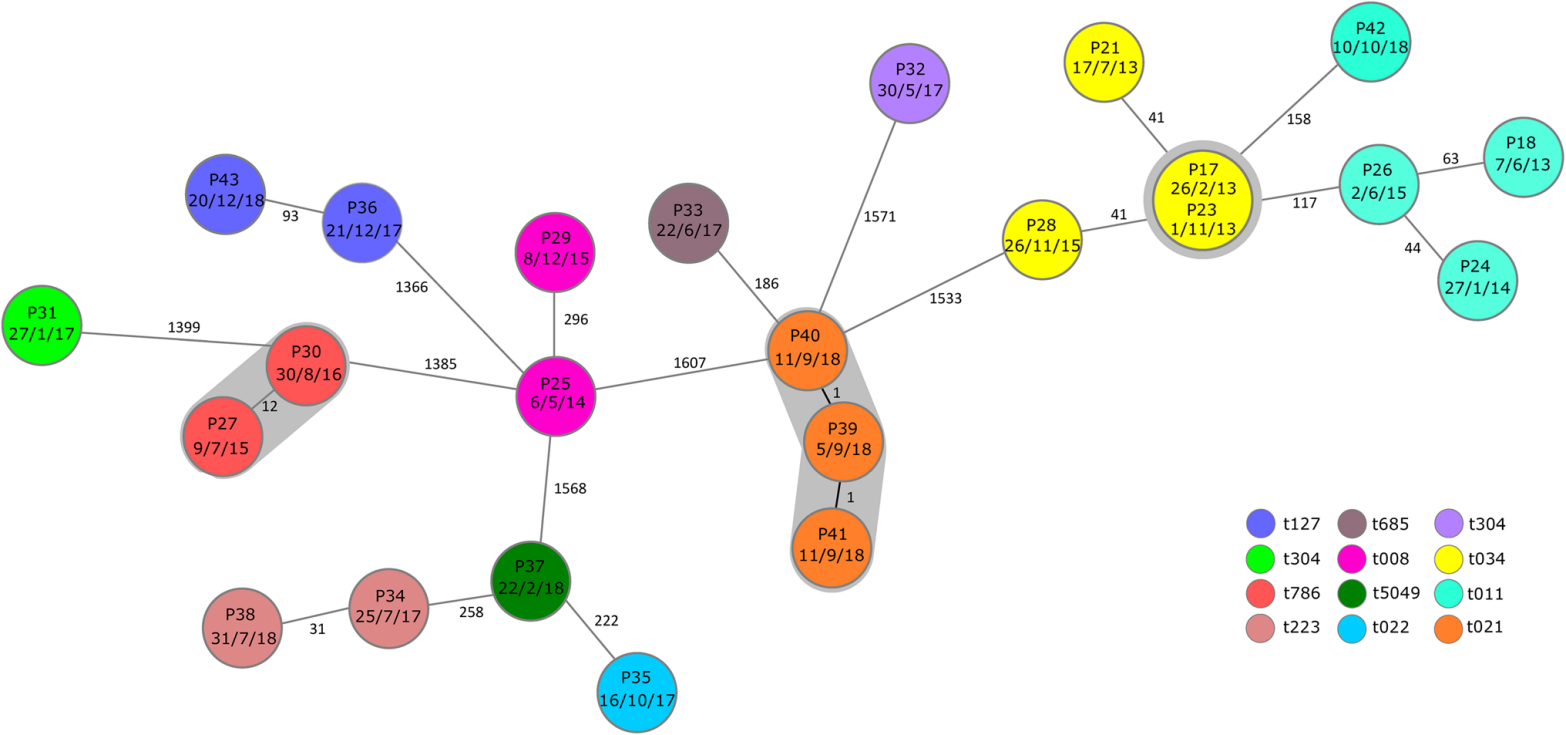
Minimum spanning tree of 24 MRSA strains (20 patient and 4 parental strains) isolated from 2013 onward from patients with cancer or allogeneic HCT and their parents, if applicable. Each circle represents a unique allele profile based on 1861 cgMLST target genes. The number refers to the patient as listed in table S1, and the date represents the date of the first isolate. Size of circles correlates with the number of identical isolates; colors of circles indicate the respective spa-type. Numbers near to the connecting lines show the number of alleles differing between two genotypes. Whole genome sequencing revealed three clusters (highlighted in gray) of MRSA, all containing isolates of individual patients and their colonized parents

Table 1 shows the resistance profiles of the initial MRSA isolates from the 34 patients and 8 parents identified to be colonized (Table 1). Of note, all isolates were susceptible to mupirocin, which is used for nasal application during decolonization. In addition, no phenotypical in vitro resistances to vancomycin, linezolid, or daptomycin, antibacterial agents commonly used for treatment of MRSA infections, was observed. Analyzing spa-types of isolated MRSA in silico from WGS data revealed t011 (22.6%) and t034 (12.9%) to be most prevalent (Table 1). All detected MRSA isolates harbored the mecA resistance gene, while genes determing PVL were detected in two isolates (Table 1). Genetic comparison of isolates from 2013 onward using the cgMLST scheme based on 1861 genes present in all isolates revealed three clusters of genetically closely related genotypes (Fig. 2), each pertaining to isolates of individual patients and their parents. Genetic comparison of all other strains showed no genetic similarities between strains isolated from patients and parents during 2013–2018, indicating that no nosocomial transmissions of one and the same clone had taken place.

**Table 1:** Antimicrobial resistance patterns of 43 initial MRSA strains isolated from 34 pediatric patients with cancer or allogeneic HCT and 8 parents found to be colonized^a^

Isolate No	MUP	CN	CIP	LEV	MOX	CLI	ERY	AZM	TET	TIG	VAN^b^	TEI	T/S	RIF	FOS	LIN	DAP^c^	*mecA*	PVL	*spa*-type
P1	R	S	S	S	S	S	S	S	S	S	S	S	S	S	S	S	NT	+	-	t645
P2	R	S	S	S	S	S	S	S	S	S	S	S	S	S	S	S	NT	+	-	t449
P3	R	S	S	S	S	S	S	S	S	S	S	S	S	S	S	S	NT	+	-	t044
P4	R	S	S	R	R	R	S	R	R	S	S	S	S	S	S	S	NT	+	-	t003
P5	R	S	S	S	S	S	R	R	R	R	S	S	S	R	S	S	NT	+	-	t034
P6	R	S	S	S	S	S	R	R	R	R	S	S	S	R	S	S	NT	+	-	t034
P7	R	S	S	S	S	S	R	S	S	R	S	S	S	S	S	S	NT	+	-	t108
P8	R	S	S	S	S	S	S	S	S	S	S	S	S	S	S	S	NT	+	-	t011
P9	R	S	S	S	S	S	S	S	S	S	S	S	S	S	S	S	NT	+	-	t019
P10	R	S	S	R	R	R	R	R	R	S	S	S	S	S	S	S	NT	+	-	t003
P11	R	S	S	S	S	S	R	R	R	R	S	S	S	R	S	S	NT	+	-	t113
P12	R	S	S	S	S	S	R	R	R	R	S	S	S	S	S	S	NT	+	-	t034
P13	R	S	S	R	R	R	R	S	S	R	S	S	S	R	S	S	NT	+	-	t003
P14	R	S	S	S	S	S	S	S	S	S	S	S	S	S	S	S	NT	+	-	t002
P15	R	S	S	S	S	S	S	S	S	S	S	S	S	S	S	S	S	+	-	t223
P16	R	S	S	S	S	S	R	R	R	R	S	S	S	S	S	S	S	+	-	t127
P17	R	S	S	S	S	S	R	R	R	R	S	S	S	R	S	S	S	+	-	t034
P18	R	S	S	S	S	S	R	R	R	R	S	S	S	R	S	S	S	+	-	t011
P19	R	S	S	S	S	S	R	R	R	R	S	S	S	R	S	S	S	+	-	t011
P20	R	S	S	S	S	S	S	S	S	R	S	S	S	S	S	S	NT	+	-	t011
P21	R	S	S	S	S	S	R	R	R	R	S	S	S	R	S	S	S	+	-	t034
P22	R	S	S	S	S	S	R	R	R	R	S	S	S	R	S	S	S	+	-	t011
P23	R	S	S	S	S	S	R	R	R	R	S	S	S	R	S	S	S	+	-	t034
P24	R	S	S	S	S	S	R	R	R	R	S	S	S	S	S	S	NT	+	-	t022
P25	R	S	S	S	S	S	S	S	S	S	S	S	S	S	S	S	NT	+	+	t127
P26	R	S	S	S	S	S	S	S	S	R	S	S	S	S	S	S	S	+	-	t011
P27	R	S	S	S	S	S	S	S	S	S	S	S	S	R	S	S	S	+	-	t786
P28	R	S	S	S	S	S	R	R	R	R	S	S	S	R	S	S	S	+	-	t034
P29	R	S	S	S	S	S	S	S	S	S	S	S	S	S	S	S	S	+	-	t008
P30	R	S	S	S	S	S	S	S	S	S	S	S	S	S	S	S	S	+	-	t786
P31	R	S	S	S	S	S	S	S	S	S	S	S	S	S	S	S	S	+	-	t304
P32	R	S	S	S	S	S	S	S	S	R	S	S	S	S	S	S	S	+	+	t314
P33	R	S	S	S	S	S	R	R	R	R	S	S	S	S	S	S	S	+	-	t685
P34	R	S	S	S	S	S	S	S	S	S	S	S	S	S	S	S	S	+	-	t223
P35	R	S	S	R	R	R	R	R	R	R	S	S	S	S	S	S	S	+	-	t022
P36	R	S	S	S	S	S	R	R	R	R	S	S	S	S	S	S	S	+	-	t127
P37	R	S	S	S	S	S	R	R	R	R	S	S	S	S	S	S	S	+	-	t5049
P38	R	S	S	S	S	S	S	S	S	S	S	S	S	R	S	S	S	+	-	t223
P39	R	S	S	S	S	S	S	S	S	S	S	S	S	S	S	S	S	+	-	t021
P40	R	S	S	S	S	S	S	S	S	S	S	S	S	S	S	S	S	+	-	t021
P41	R	S	S	S	S	S	S	S	S	S	S	S	S	S	S	S	S	+	-	t021
P42	R	S	S	S	S	S	R	R	R	R	S	S	S	R	S	S	S	+	-	t011
P43	R	S	S	S	S	S	R	R	R	R	S	S	R	S	S	S	S	+	-	t127

^a^Including the isolate of one non-cancer patient admitted to the oncology inpatient ward for logistic reasons (only available free bed in the oncology inpatient unit on that day) for < 24 h not included in the remaining analyses

^b^MIC values for vancomycin were available for 32 strains. Of these, the MIC was 0.5 mg/l or lower in 13, 1 mg/l in 16, and 2 mg/l in three isolates, respectively

^c^Routine testing was not established prior to 2013 (only 26 isolates tested)

*MUP* Mupirocin, *CN* Gentamicin, *CIP* Ciprofloxacin, *LEV* Levofloxacin, *MOX* Moxifloxacin, *CLI* Clindamycin, *ERY* Erythromycin, *AZM* Azithromycin, *TET* Tetracycline, *TIG* Tigecycline, *VAN* Vancomycin, *TEI* Teicoplanin, *T/S* Trimethoprim/sulfamethoxazole, *RIF* Rifampicin, *FOS* Fosfomycin, *LIN* Linezolid, *DAP* Daptomycin

### Efficacy of MRSA decolonization and time spent in isolation

Implementation of the standard MRSA decolonization regimen (see also Sect. 2.3) led to successful and lasting long-term decolonization after one (15; 57.7%), two (8, 30.8%) or more than 2 (3; 11.5%) attempts in 26 of the 34 patients (76.5%). Four patients had relapsing colonization despite multiple decolonization courses. Two patients died prior to completion of follow-up, and two patients were lost to follow-up. All MRSA-positive patients accumulated a total of 224 inpatients days spent in single room isolation, accounting to 0.3% of all inpatient days during the study period.

### Incident invasive MRSA infections

During the 12-year study period, two patients presented with invasive MRSA infections (0.15 per 1000 admission/0.03 per 1000 inpatient days). Patient 24 presented with a documented Broviac central venous catheter infection with bacteremia and patient 25 with a wound infection post Broviac central venous catheter implantation. Both patients were found to have nasopharyngeal colonization upon inpatient admission. In both patients, the infection was cured with appropriate source control (removal of the catheter and decolonization) and antibacterial therapy according to the antibiotic susceptibility testing.

## Discussion

To control MRSA transmission and emergence of MRSA infections, screening, isolation, and decolonization in all inpatient admissions were implemented at our institution from 2007 onward. The results of the present audit covering the time between 2007 and 2018 demonstrate a consistently low prevalence of colonization among incoming patients and their relatives, only sporadic infections, and lasting decolonization in the majority of patients, indicating the functioning of the implemented infection control strategy in the high-risk setting of pediatric cancer and HCT patients.

WGS data and cgMLST algorithm revealed no close genetic relatedness between MRSA isolates originating from inpatients. An absence of nosocomial transmissions during the study period can therefore be considered. In silico extraction of WGS data and previous spa-typing data resulted in 20 different spa-types, of which t034 and t011 were most prevalent. This very much reflects the local MRSA epidemiology comprising a high proportion of livestock-associated MRSA. Spa-type t008, which can be associated to community-associated USA300 clones harboring PVL genes [17], was detected twice. Interestingly, no PVL-coding gene could be detected in these strains, but in two other isolates, of which one lead to an invasive infection in patient 25. The second PVL-positive isolate did not result in an invasive infection, an observation that underlines the relevance of host–pathogen interactions in the pathogenesis of MRSA infections.

Although implemented in many healthcare systems, controversy exists concerning the optimal handling of MRSA screening. Here, targeted vs. overall screening strategies can come into play [18–21]. In particular in populations with low MRSA-rates, the amount of screening necessary to avoid infections or transmissions is very high and may have low clinical benefit while causing relevant cost; this low efficiency may be further enhanced by the low likelihood of infections following colonization in low-risk patients [22]. However, as pediatric patients with cancer or following allogeneic HCT present with and accumulate risk factors for progression of colonization to infection and undergo repeat episodes of profound granulocytopenia, screening for MRSA colonization and early decolonization should be beneficial to prevent infection and its associated morbidity and mortality. This is in particular important as the overall in-hospital MRSA prevalence per 1000 hospital days at the University Hospital of Muenster showed a consistent decline (1.44–0.65) during the observed time span, whereas the MRSA prevalence in the present patient cohort did not follow this trend. Nevertheless, the question whether the quality of care would severely be influenced if only targeted testing was applied cannot be answered definitely. This study gives a first hint that overall screening for surveillance of MRSA and as a part of a prevention bundle strategy is desirable in a low prevalence setting.

Nasal carriage is known to play an important role in the development of MRSA infections. Several studies have shown that the majority of bloodstream isolates were clonal to the ones found previously in nasal swabs [23, 24] and an increased risk of infection in MRSA colonized patients of neonatal and pediatric ICU children has been well-documented [25]. Despite this correlation, the question of whether and how to prevent bloodstream infections by decolonizing patients with positive nasal swabs remains a matter of discussion. In a systematic Cochrane review, Van Rijen et al. analyzed nine randomized controlled trials (RCTs) with a total of 1690 patients undergoing mupirocin treatment for nasal MRSA carriage. After pooling those RCTs, there was a significant reduction of S. aureus infections [26]. Similar results have been obtained in multicenter studies in children, especially for neonatal intensive care unit (NICU)-patients [27]. Huang et al. even proposed the use of universal decolonization for ICU-patients without first testing them for MRSA, since it may avoid the delay of intervention while waiting for the screening results [28].

Decolonization therapy failed in 11.8% (4/34) of our patients without detectable correlation to the underlying disease (acute undifferentiated leukemia, sickle cell disease, nephroblastoma and Ewing sarcoma; each *n* = 1), migration background (*n* = 1), or colonization of other family members (*n* = 1). In a systematic review of twenty-three studies, Ammerlaan et al*.* described a long-term decolonization rate of 60% [29]. With a median follow-up of 32 months (range 0–127 months), we recorded a slightly higher decolonization rate of 76.5%, that, however, might be affected by the small sample size of our study.

Consequent hand hygiene is considered to be the most important intervention within the bundle of contact precautions. Giuffè et al. showed a reduced transmission of multidrug-resistant bacteria in a neonatal intensive care unit after implementation of strict hand hygiene standards [30]. In contrast, the beneficial effects of other measures including patient isolation and the additional use of gloves, gowns, and masks still need further investigation [31, 32]. Indeed, since most precautions for transmission control are performed in bundles of interventions, future studies need to address evidence of single actions.

There were two invasive MRSA infections during the 12 years of observation. The first was a local wound infection at a Broviac catheter exit site that was successfully treated by surgery and topic therapy; nasopharyngeal colonization was detected upon inpatient admission for surgery. The second infection was a catheter-associated blood stream infection in a high-risk patient after allogeneic HCT. The low rate of additional resistances in the MRSA isolates in the study presented here may have had an influence on the mostly successful decolonization and therapy of the infections that occurred. All isolates were found to be sensitive to mupirocin, which is the standard antibiotic used for decolonization. Until 2014, standard antibacterial therapy for fever during granulocytopenia consisted of combination therapy with gentamicin, which was effectively tested for all isolates. Escalation in critically ill or persistently febrile patients included a glycopeptide which were also active against all isolates collected from the 34 patients. As a consequence, given the current resistance situation, all patients will promptly receive empirical treatment with an antibacterial agent that is effective against MRSA so that the use of second line reserve agents is not necessary at present for successful MRSA management in our institution.

The present study has limitations. First, given the overall low MRSA prevalence rates in our hospital compared to other hospitals and countries, generalization of present results might be restricted. International multicenter studies will be necessary to further underline our observations in higher prevalence settings. Second, as we directly implement bundle strategies including rapid MRSA identification and decolonization, effects of one single measure could not be figured out in the present investigation. Nevertheless, this approach reflects the current standard of care.

In conclusion, we observed a low rate of MRSA colonization and infection during a 12-year time period after universal implementation of screening, isolation, and decolonization. These low rates and the absence of nosocomial patient-to-patient transmission suggest the effectiveness of the management bundle of MRSA identification, isolation, and decolonization in the vulnerable population of pediatric patients with cancer or allogenic HCT in a low prevalence setting.

## Supplementary Information

Below is the link to the electronic supplementary material.

Supplementary file1 (DOCX 22 KB)

## Data Availability

All data generated or analyzed during this study are included in this published article or available from the corresponding author on reasonable request.
