# Supplementary material for: Prospective surveillance of colonization and disease by methicillin-resistant Staphylococcus aureus (MRSA) at a European pediatric cancer center
Source: Support Care Cancer. 2022 May 19;30(9):7231–9. doi: 10.1007/s00520-022-07140-0 (PMC9385780; doi:10.1007/s00520-022-07140-0)
Supplement: Supplementary file 1 — Supplementary file1 (DOCX 22 KB) [file 520_2022_7140_MOESM1_ESM.docx]

**Prospective Surveillance of Colonization and Disease by Methicillin-Resistant *Staphylococcus aureus* (MRSA) at a European Pediatric Cancer Center**

Miriam A. Füller^1#^, Stefanie Kampmeier^2#^ (ORCiD: 0000-0002-5013-159X), Anna M. Wübbolding^1^, Judith Grönefeld^1^, Almut Kremer^3^, Andreas H. Groll^1^*

^#^ both authors contributed equally

^1^ Infectious Disease Research Program, Center for Bone Marrow Transplantation and Department of Pediatric Hematology and Oncology, University Children’s Hospital Münster, Münster, Germany

^2^ Institute of Hygiene, University Hospital Münster, Münster, Germany

^3^ Medical Controlling, University Hospital Münster; Münster, Germany

**Corresponding author:*

Andreas H. Groll, Infectious Disease Research Program, Center for Bone Marrow Transplantation and Department of Pediatric Hematology and Oncology, University Children’s Hospital Münster, Münster, Germany; andreas.groll@ukmuenster.de

**Table S1**: Demographic characteristics, risk factors, isolation burden, eradication and colonization and infection characteristics of pediatric patients with cancer or allogeneic HCT and parents presenting with positive MRSA testing or infection.

| **Isolate No.** | **Patient No.** | **Year** | **Age (years)** | **Gender** | **Diagnosis** | **Migration background** | **Type of surgery** | **Central line** | **Antibiotics (<4 weeks)** | **S/p allo HCT** | **Family involvement** | **Colonization (nose/throat)** | **Colonization (axilla/groin)** | **Time to eradication (days)** | **Isolation (days)** | **Invasive infection** | **Farming background** |
| --- | --- | --- | --- | --- | --- | --- | --- | --- | --- | --- | --- | --- | --- | --- | --- | --- | --- |
| P1 |  | 2007 | Patient with metabolic disorder admitted for logistic reasons for <24 h | | | | | | | | | | | | | | |
| P2 | 1 | 2007 | 17.7 | f | ALL | Italy | - | - | T/S | yes | - | yes | nt | l.t.f. | 3 | - | - |
| P3 | 2 | 2007 | 2.7 | m | ALL | - | - | Port | - | - | - | yes | nt | 7 | 7 | - | yes |
| P4 | 3 | 2008 | 2.2 | f | ALL | - | - | Port | T/S | - | - | yes | nt | 9 | 7 | - | - |
| P5 | 4 | 2008 | 17.1 | m | ALL | - | abscess incision; scrotal biopsy | Port | T/S | - | - | yes | nt | 65 | 0 | - | - |
| P6 | 5 | 2009 | 1.75 | f | EWS | - | tumor biopsy/ resection | Broviac | T/S | - | - | yes | yes | 29 | 11 | - | - |
| P7 | 6 | 2009 | 2.25 | m | WT | - | circumcision | Port | T/S | - | - | yes | yes | no | 5 | - | yes |
| P8 | 7 | 2010 | 6.0 | m | HL | - | scrotal biopsy | - | - | - | yes | yes | nt | 5 | 14 | - | yes |
| P9 |  | 2010 | Incidental finding, parent of a MRSA negative HCT patient | | | | | | | | | | | | | | |
| P10 | 8 | 2011 | 16.0 | f | OS | - | - | Port | T/S, CFZ, GEN | - | - | yes | nt | 5 | 4 | - | - |
| P11 | 9 | 2011 | 8.25 | m | AML | - | tooth extraction | Broviac | T/S, CFX, GEN, CFZ,TEI, MPM, MDZ | - | - | yes | nt | no | 23 | - | - |
| P12 | 10 | 2011 | 14.1 | f | LA | - | multiple resections; gastro-and tracheostomy | - | - | - | - | yes | yes | 1422 | 0 | - | - |
| P13 | 11 | 2011 | 1.1 | f | EP | - | tumor biopsy/ resection; VP shunt | Port | T/S | - | - | yes | yes | 31 | 9 | - | - |
| P14 | 12 | 2012 | 16.8 | m | AML | - | - | - | - | yes | - | yes | - | 71 | 3 | - | - |
| P15 | 13 | 2012 | 12.9 | m | EWS | Russia | - | Port | - | - | - | yes | - | 17 | 25 | - | - |
| P16 |  | 2012 | Incidental finding, parent of a MRSA negative HCT patient | | | | | | | | | | | | | | |
| P17 | 14 | 2013 | 0.1 | f | NB | - | tumor biopsy | Port | - | - | yes | yes | - | 904 | 11 | - | - |
| P18 | 15 | 2013 | 16.9 | m | ALL | - | - | Port | T/S | - | yes | yes | - | 15 | 16 | - | yes |
| P19 | 16 | 2013 | 10.8 | m | AC | - | - | Port | T/S | - | - | yes | - | 333 | 0 | - | - |
| P20 |  | 2013 | Parent (P18) | | | | | | | | | | | | | | |
| P21 | 17 | 2013 | 16.6 | m | EWS | - | - | Port | T/S | - | - | yes | - | no | 0 | - | - |
| P22 | 18 | 2013 | 17.5 | m | ALL | - | Rickham removal, ventricular drainage | Broviac | T/S | yes | - | yes | - | 50 | 4 | - | - |
| P23 |  | 2013 | Parent (P17) | | | | | | | | | | | | | | |
| P24 | 19 | 2014 | 17.1 | m | ALL | - | - | Broviac | - | yes | - | yes | yes | 1418 | 3 | yes | - |
| P25 | 20 | 2014 | 15.8 | f | AML | - | - | Broviac | T/S | yes | - | yes | - | no | 23 | yes | - |
| P26 | 21 | 2015 | 17.4 | m | GCT | - | - | CVC | - | - | - | yes | yes | 254 | 14 | - | - |
| P27 | 22 | 2015 | 1.8 | m | SCD | Nigeria | - | Port | - | - | yes | yes | yes | no | 2 | - | - |
| P28 | 23 | 2015 | 10.1 | f | CV | - | - | - | - | - | - | yes | - | 230 | 0 | - | - |
| P29 | 24 | 2015 | 15.2 | m | OS | - | thoracotomy | Port | - | - | - | yes | - | 40 | 7 | - | - |
| P30 |  | 2016 | Parent (P27) | | | | | | | | | | | | | | |
| P31 | 25 | 2017 | 12.2 | f | THAL | Syria | - | Broviac | - | yes | - | yes | - | 90 | 0 | - | - |
| P32 | 26 | 2017 | 16.6 | m | SCD | Libya | cholecystectomy | - | - | - | - | yes | - | 66 | 0 | - | - |
| P33 | 27 | 2017 | 10.0 | m | SCD | Angola | - | Broviac | - | yes | - | yes | - | 271 | 0 | - | - |
| P34 |  | 2017 | Incidental finding, parent of a MRSA negative HCT patient | | | | | | | | | | | | | | |
| P35 | 28 | 2017 | 3.8 | m | SCD | - | - | Broviac | PEN | yes | - | yes | - | 61 | 43 | - | - |
| P36 |  | 2018 | Parent (P40) | | | | | | | | | | | | | | |
| P37 | 29 | 2017 | 10.8 | m | ALL | Romania | - | Broviac | T/S; AZM | yes | - | yes | - | 65 | 2 | - | - |
| P38 | 30 | 2018 | 2.2 | m | BDA | Afghanistan | - | - | - | - | - | yes | yes | l.f.f. | 0 | - | - |
| P39 | 31 | 2018 | 1.9 | m | EP | Syria | tumor biopsy | port | T/S | - | - | yes | - | 20 | 0 | - | - |
| P40 | 32 | 2018 | 8.1 | m | AC | Armenia | tumor biopsy | port | T/S | - | yes | yes | yes | 99 | 0 | - | - |
| P41 |  | 2018 | Parent (P40) | | | | | | | | | | | | | | |
| P42 | 33 | 2018 | 21.3 | m | AUL | - | - | - | - | yes | - | yes | - | no | 0 | - | - |
| P43 | 34 | 2018 | 4.4 | m | RMS | Romania | tumor biopsy | port | CFX | - | - | yes | - | 55 | 13 | - | - |

Positive parents are greyed out. Isolate numbers correlate to those in figure 2.

AC, astrocytoma; ALL, acute lymphoblastic leukemia; AML, acute myeloid leukemia; AUL, acute undifferentiated leukemia; BDA, Blackfan Diamond Anemia; CV, cerebral vasculitis and stroke; EP, ependymoma; EWS, Ewing’s sarcoma; GCT, extracerebral germ cell tumor; HL, Hodgkin’s lymphoma; LA, lymphangioma; NB, neuroblastoma, OS, osteosarcoma; RMS, rhabdomyosarcoma; SCD, sickle cell disease; THAL, thalassemia major; WT, nephroblastoma;

T/S, trimethoprim/sulfamethoxazole; AZM, azithromycin; CFX, cefixime; CFZ; ceftazidime; GEN; gentamicin; TEI, teicoplanin, MPM, meropenem; MDZ, metronidazole; PEN, penicillin V;

n.t., not tested; l.t.f., lost to follow-up
